# P-889. Impact of an Intravenous Fluid Shortage on the Use of Targeted Oral Antimicrobials at 39 Hospitals in the Southeastern United States

**DOI:** 10.1093/ofid/ofaf695.1097

**Published:** 2026-01-11

**Authors:** Melissa D Johnson, Jeannette Bouchard, Angelina Davis, April Dyer, Elizabeth Dodds Ashley

**Affiliations:** Duke University, Durham, North Carolina; Duke Antimicrobial Stewardship Outreach Network, Elgin, SC; Duke Center for Antimicrobial Stewardship and Infection Prevention, Durham, NC; Duke Center for Antimicrobial Stewardship and Infection Prevention, Durham, NC; Duke Center for Antimicrobial Stewardship and Infection Prevention, Durham, NC

## Abstract

**Background:**

Administration of intravenous (IV) antimicrobials often requires availability of IV fluid minibags. Hurricane Helene severely impacted a major manufacturing plant in North Carolina in Sept 2024, leading to a critical IV fluid shortage. Mitigation strategies included relying on increasingly available data to support use of oral (PO) antimicrobials for serious infections. We aimed to assess the impact of the IV fluid shortage on use of PO formulations of highly bioavailable antimicrobials in community hospitals in the Southeastern US participating in the Duke Antimicrobial Stewardship Outreach Network (DASON).

**Table.**
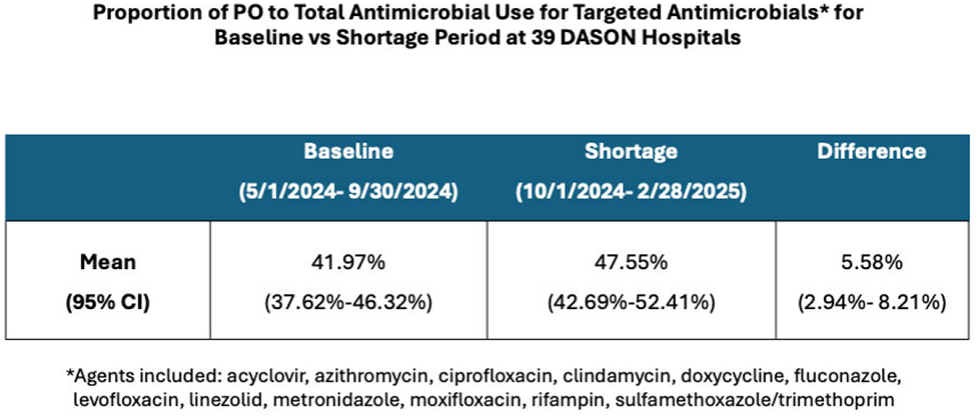
Proportion of PO to Total Antimicrobial Use for Targeted Antimicrobials for Baseline vs Shortage Period at 39 DASON Hospitals

**Fig 1.**
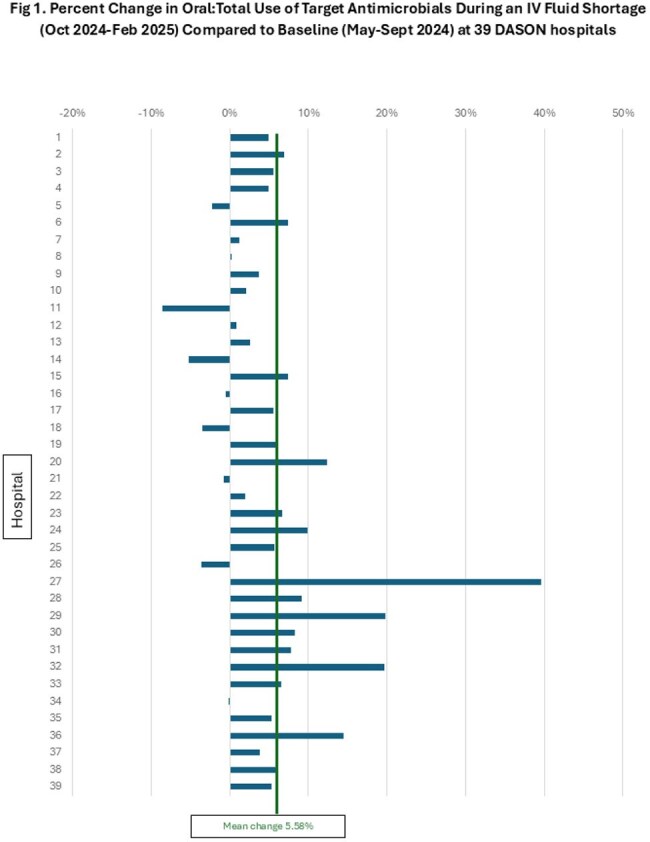
Percent Change in Oral:Total Use of Target Antimicrobials During an IV Fluid Shortage (Oct 2024-Feb 2025) Compared to Baseline (May-Sept 2024) at 39 DASON hospitals

**Methods:**

We performed a retrospective analysis of antimicrobial use (AU) on inpatient units of 39 DASON hospitals from 5/1/2024-9/30/24 (“Baseline Period”) compared to 10/1/2024-2/28/2025 (“Shortage Period”). AU for PO and IV formulations of 12 antimicrobials were compared (Wilcoxon Signed Rank Test) for the two time periods using Days of Therapy (DOT)/1,000 patient days for each hospital, with percent change of PO:Total AU calculated for each hospital.

**Fig 2.**
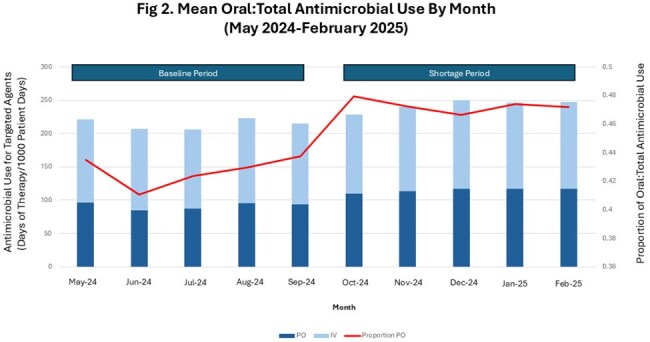
Mean Oral:Total Antimicrobial Use By Month (May 2024-February 2025)

**Results:**

We identified 341,622 days of antimicrobial therapy for targeted antibiotics at 39 hospitals from 5/1/2024-2/28/2025. During the Baseline Period, PO formulations accounted for 41.97% of administrations which increased to 47.55% during the Shortage Period, with a mean difference of 5.58% (Wilcoxon Signed Rank p< 0.0001) (Table). The proportion of PO:Total AU for these agents varied across hospitals (Fig 1), with substantial increases at some facilities while others decreased. Overall, monthly AU data (Fig 2) showed a steep increase in proportion of PO:Total AU beginning with the IV fluid shortage in Oct 2024, which was sustained throughout the Shortage Period.

**Conclusion:**

The IV fluid shortage experienced in the aftermath of Hurricane Helene impacted use of highly bioavailable antimicrobials in a network of 39 hospitals in the Southeastern US. This led to a shift in the use of PO formulations of these agents, with a mean increase of 5.58% across the network. Shifts in AU varied across the hospitals, suggesting differences in impact and response to the shortage at different facilities. Additional surveillance is needed to evaluate the overall impact of this event after the shortage resolved in March 2025.

**Disclosures:**

Melissa D. Johnson, PharmD MHS AAHIVP, Biomeme: Licensed technology, method to detect fungal infection|Biomeme: Licensed technology, method to detect fungal infection|Scynexis: Grant/Research Support|Scynexis: Grant/Research Support|UpToDate: Author Royalties|UpToDate: Author Royalties Elizabeth Dodds Ashley, PharmD, MHS, HealthtrackRx: Advisor/Consultant|UpToDate, Inc.: Author Royalties

